# Gene Interaction of Dopaminergic Synaptic Pathway Genes in Attention-Deficit Hyperactivity Disorder: a Case-Control Study in Chinese Children

**DOI:** 10.1007/s12035-023-03523-4

**Published:** 2023-08-14

**Authors:** Lin Zhong, Hongyao He, Jing Zhang, Xiaoyan Gao, Feifei Yin, Pengxiang Zuo, Ranran Song

**Affiliations:** 1 Medical College of Shihezi University, Xinjiang Shihezi, 832000 China; 2 Department of Maternal and Child Health and MOE Key Lab of Environment and Health, School of Public Health, Huazhong University of Science and Technology, Wuhan, 430000 China

**Keywords:** Attention-deficit hyperactivity disorder, Dopaminergic synapse pathway genes, DRD2, SLC6A3, GRIN2B, Interaction

## Abstract

Attention-deficit hyperactivity disorder is a highly inherited neurodevelopmental disorder. Previous genetic research has linked ADHD to certain genes in the dopaminergic synaptic pathway. Nonetheless, research on this relationship has produced varying results across various populations. China is a multi-ethnic country with its own unique genetic characteristics. Therefore, such a population can provide useful information about the relationship between gene polymorphisms in dopaminergic synaptic pathways and ADHD. This study looked at the genetic profiles of 284 children in China’s Xinjiang. In total, 142 ADHD children and 142 control subjects were enrolled. Following the extraction of DNA from oral mucosal cells, 13 SNPs for three candidate genes (SLC6A3, DRD2, and GRIN2B) in the dopaminergic synaptic pathway of ADHD were screened. Based on the results of single nucleotide polymorphism (SNP) analyses, we found that the DRD2 gene variants rs6277 and rs6275, and the SLC6A3 gene variant rs2652511, were significantly associated with ADHD in boys and girls, respectively, after adjusting for false discovery rate (FDR) in terms of allele frequencies. Furthermore, our generalized multifactorial downscaling approach identified a significant association between rs6275 and rs1012586. These findings suggest that DRD2 and SLC6A3 genes have a crucial role in ADHD susceptibility. Additionally, we observed that the interaction between GRIN2B and DRD2 genes may contribute to the susceptibility of Chinese children with ADHD.

## Introduction

Attention deficit hyperactivity disorder (ADHD) is a chronic neurodevelopmental disease that manifests as age-inappropriate hyperactivity, attention deficit, and impulsivity in children. With normal or near- normal intelligence, attention deficit symptoms usually persist into adulthood [1]. ADHD is a common neurodevelopmental disorder in children and adolescents, affecting 63 million children and adolescents worldwide [2]. The prevalence varies little across countries and regions but is influenced by various diagnostic criteria and assessment methods. The current prevalence of ADHD in children in the United States is 5–10% [3, 4] with a prevalence of 6.26 percent in Chinese children. Although symptoms fade as children grow older, some persist and can have an impact on adulthood. Therefore, ADHD has become a common public health issue, causing long-term harm to children's social functioning [5, 6] and mental health status [7]. It can also have more serious negative effects on both families and society, as well as a significant economic and mental health medical burden [8, 9]. The etiology and mechanisms of ADHD, however, remain unknown, and the influencing factors are complex [10]. Scholars generally agree on the importance of genetic factors in the etiology of childhood ADHD and assess its heritability at 76% [11]. ADHD is a complex psychiatric disorder with unknown genetic influences and mechanisms of action. Numerous genetic studies have revealed that ADHD pathogenesis may be caused by multiple genes rather than just one [12].

**Fig. 1:**
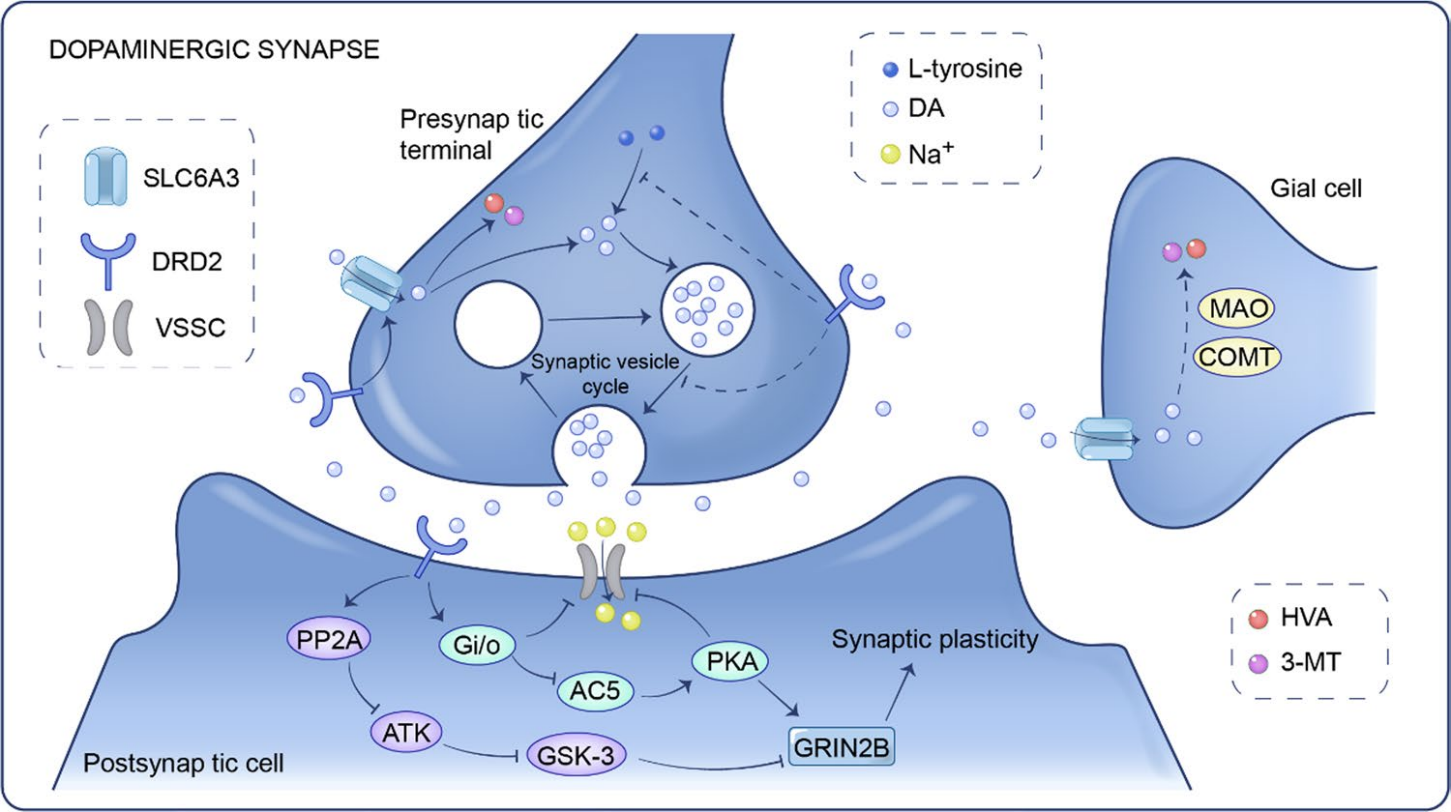
A map of dopaminergic synaptic pathways

Dopamine (DA) serves as a critical neurotransmitter within the central nervous system (CNS). It participates in the regulation of numerous physiological functions within the CNS. These include cognitive processes, focus, learning and memory consolidation, motor functions, motivational states and reward, along with mood modulation. The dopaminergic synaptic pathway's primary function is the synthesis, release, and transmission of DA, which activates receptor signaling pathways to affect synaptic plasticity (Fig. 1). Dopaminergic synaptic pathway genes (Dopamine receptor D2 (DRD2) [13, 14], Catechol-o-methyltransferase (COMT) [15, 16], Solute carrier family 3 member 6 (SLC6A3) [17], and others) are currently linked to ADHD [18]. Because DA is important for various physiological activities such as motor activity, cognition, and attention, the relationship between DA-related genes (mostly transporters and receptors) and ADHD has been studied extensively around the world. Nonetheless, the cause of ADHD is unknown. DRD2 can suppress adenylate cyclase activity as a G protein-coupled receptor, but it is currently thought to be a possible ADHD as well as a disease-related gene. Some academics, however, disagree with this viewpoint. Rowe et al. [19] found that DRD2 (particularly Taq1 polymorphism) was unrelated to ADHD, which is consistent with the findings of Kirley et al. [20].

SLC6A3, a dopamine transporter gene, has been linked to ADHD in previous studies [21]. The SLC6A3 gene encodes the dopamine transporter, which functions to return extracellular DA into the cytoplasm of presynaptic DA neurons, limiting the duration of synaptic activity. SLC6A3 is a candidate gene for ADHD that has received a lot of attention. SLC6A3 was found to be significantly correlated with ADHD in related gene research. Researchers from various countries attempted to replicate this result using various methods [22]. Approximately half of the researchers received positive results [23, 24], while the other half received no results [25, 26]. According to Brookes et al. [27], four SLC6A3 gene SNPs (rs2550946, rs2652511, rs550948, rs11564750) were linked to ADHD in children. However, a recent study calls the relationship between SLC6A3 gene polymorphisms and ADHD into question, pointing to the impact of the SLC6A3 10R genotype on attentional/cognitive functions, which deficits are not the key symptoms in ADHD [28].

Much of the molecular genetic research on ADHD has concentrated on genes related to the dopamine neurotransmitter pathway, and the glutamatergic system has been involved in ADHD pathophysiology and treatment [29]. Glutamate accounts for a main CNS excitatory neurotransmitter, which regulates neighboring neuronal activity by combining with the ionotropic glutamate receptors (iGluRs) or metabolic glutamate receptors (mGluRs). The N-methyl-D-aspartate receptor(NMDAR) belongs to the iGluR family, which has aroused wide attention for ADHD because it has an important effect on cognitive ability and prefrontal cortex (PFC) activity, like reversal learning, attention, and working memory [30, 31]. Research shows that children who possess de-novo GRIN2B mutations or intellectual disabilities are associated with impulsivity, hyperactivity, decreased attention duration, and distractibility [32]. According to family-based research, the biased transmission of rs2284411 with NMDA receptor 2B (NR2B) subunit gene (GRIN2B) polymorphisms is most significantly related to ADHD [33]. Nonetheless, there are studies reporting unfavorable findings, and large whole-genome association research has not been able to identify any obvious relation [34, 35]. Thus, further investigation is warranted to explore the association between ADHD and polymorphisms of the GRIN2B gene in Chinese children.

However, the lack of replicated genetic outcomes is due to the dysfunction of one gene, which is probably insufficient to induce ADHD, while multiple genes and/or the corresponding interactions possibly have important effects. Given the complex interactions between the glutamatergic and dopaminergic networks, the glutamatergic system, especially via NMDAR, has been suggested to participate in ADHD pathophysiology [34].Thus, internal genetic variants of glutamatergic/dopaminergic neurotransmission may affect ADHD risk or its phenotype. Transmission between dopaminergic and glutamatergic energy simultaneously acts in the dopaminergic synaptic pathway. In contrast, DRD2, SLC6A3, and GRIN2B are involved in the composition of the dopaminergic synaptic pathway and play crucial roles in it. Many investigators have now paid attention to the dopaminergic and glutamatergic systems during ADHD pathophysiology, but there is no related research examining the relationship between dopaminergic synaptic pathway genes and gender-specific ADHD susceptibility in Chinese children.

Over the last decade, single nucleotide polymorphisms (SNPs) have been used to examine genetic influences on ADHD susceptibility, which has increasingly provided important scientific evidence to unravel the mechanisms and individual differences in the development of ADHD. However, as the number of related studies increased, researchers began to question the validity of individual SNP studies. Individual SNP-based candidate gene studies have low statistical power and produce widely disparate results across populations [36]. Multiple SNPs' combined and cumulative effects were more closely associated with complex phenotypes of ADHD than single SNPs [37]. Therefore, an in-depth examination of the relationship between genes has become an effective method of studying the genetic mechanisms of ADHD. Davies' study revealed that males are 2–4 times more likely than females to meet the diagnostic criteria for ADHD in children, and that gender also influences the severity of morbidity and complications [38]. A recent FMRI study reported reduced volume in the putamen and thalamus in girls with ADHD and suggested gender dimorphism in neuroanatomical development in children with ADHD [39]. These findings indicate that genetic susceptibility to ADHD varies by gender and emphasize the importance of investigating such differences. Therefore, it is crucial to explore the genetic vulnerability to ADHD in the context of gender disparities. As a result of the low genetic drift and population mobility, such people have specific genetic profiles in terms of molecular gender-specific genetics for various disorders [40]. Therefore, given the genetic differences among global populations, the present study selected 13 SNPs (rs1124491, rs1079727, rs6275, rs6277, rs6278, rs2652511, rs2975226, rs6347, rs1012586, rs1805502, rs1806191, rs2268119, rs7301328) to comprehensively analyze the genetic pathogenesis of ADHD in children by studying the cumulative effects of multiple genes based on dopaminergic synaptic pathways and gene-gene interaction studies.

## Results

This study enrolled a total of 284 subjects, including 142 children in the ADHD group and 142 children in the control group. Table 1 displays the subject’s general features. Children with ADHD and healthy controls were paired according to age, sex, IQ score, and education (*P* > 0.05).

**Table 1:** General characteristics of the study population

Variable	Case (*N* = 142)^a^	Control (*N* = 142)^a^	χ^2^/t	*P*^b^
Gender				
Male	97(68.3)	98(69.0)	0.016	0.898
Female	45(31.7)	44(31.0)		
Age	10.56 ±1.573	10.33 ±1.552	- 1.253	0.211
IQ score	96.98 ±9.009	98.49 ±8.441	1.462	0.145

^a^Data are represented by *n* (%) or mean ±SD

^b^*P*-values were analyzed through Student’s *t*-test or chi-square test for continuous and categorized variables, separately

After FDR multiple test correction, genotype analysis revealed that the DRD2 gene rs6277, rs6275, and SLC6A3 gene rs2652511 had distinct heterogeneity in the distribution of ADHD and healthy subjects (*P*< 0.05) (Table 2). Under the dominant model, we discovered that DRD2 gene rs6277 had a 1.94 times higher ADHD risk in children with GA and AA genotypes than in children with GG genotype (OR = 1.94, 95% CI = 1.20–3.16). However, this association was only significant in males and not in females. The ADHD risk in males who carried GA and AA genotypes was 2.95 times higher (OR = 2.95, 95% CI = 1.57–5.53) than in children carrying the GG genotype for the DRD2 gene rs6277. The ADHD risk in children who carried AG and AA genotypes was 2.69 times higher (OR = 2.69, 95% CI = 1.51–4.79) than in children carrying the GG genotype for the DRD2 gene rs6275. However, this association was significant only in males, where the ADHD risk was 3.35 times higher in those carrying AG and AA genotypes (OR = 3.35, 95% CI = 1.59–7.03) than in those carrying GG genotype for the DRD2 gene rs6275. Children carrying the AG and GG genotypes had a 0.49-fold risk of ADHD in comparison with those who carried the AA genotype (OR = 0.49, 95% CI = 0.31–0.80) for the SLC6A3 gene rs2652511. However, this association was significant only in females, where the ADHD risk was 0.41 times lower in those carrying AG and AA genotypes (OR = 0.41, 95% CI = 0.17–0.96) than in those carrying the GG genotype (Table 2).

**Table 2:** Allele frequencies and genotype distributions in patients with ADHD and in normal controls, segmented by gender

Gene	SNP	Model	Genotype	Total			Male			Female		
				Control/Case	OR (95%CI)	*P*	Control/Case	OR (95%CI)	*P*	Control/Case	OR (95%CI)	*P*
DRD2	rs1124491	Codominant	G/G	79/69	1	0.27	49/49	1	0.89	30/20	1	0.098
			A/G	48/60	1.43(0.87–2.36)		36/41	1.14 (0.63–2.07)		12/19	2.37 (0.95–5.95)	
			A/A	5/8	1.83(0.57–5.86)		4/5	1.25 (0.32–4.93)		1/3	4.50(0.44–46.38)	
		Dominant	G/G	79/69	1	0.12	49/49	1	0.64	30/20	1	0.037
			A/G-A/A	53/68	1.47(0.91–2.38)		40/46	1.15 (0.64–2.05)		13/22	2.54 (1.04–6.18)	
		Recessive	G/G-A/G	127/129	1	0.43	85/90	1	0.81	42/39	1	0.28
			A/A	5/8	1.58(0.50–4.94)		4/5	1.18 (0.31–4.54)		1/3	3.23(0.32–32.38)	
		Overdominat	G/G-A/A	84/77	1	0.21	53/54	1	0.71	31/23	1	0.096
			A/G	48/60	1.36(0.84–2.22)		36/41	1.12 (0.62–2.01)		12/19	2.13 (0.87–5.26)	
	rs6277	Codominant	G/G	87/71	1	0.025*	70/52	1	0.0017*	17/19	1	0.74
			G/A	40/64	1.96 (1.18–3.25)		17/41	3.25 (1.66–6.34)		23/23	0.89 (0.37–2.14)	
			A/A	6/9	1.84 (0.62–5.14)		4/5	1.68 (0.43–6.58)		2/4	1.79(0.29–11.04)	
		Dominant	G/G	87/71	1	0.006*	70/52	1	0.00005*	17/19	1	0.94
			G/A-A/A	46/73	1.94 (1.20–3.16)		21/46	2.95 (1.57–5.53)		25/27	0.97 (0.41–2.26)	
		Recessive	G/G-G/A	127/135	1	0.52	87/93	1	0.82	40/42	1	0.46
			A/A	6/9	1.41 (0.49–4.08)		4/5	1.17 (0.30–4.50)		2/4	1.90(0.33–10.98)	
		Overdominat	G/G-A/A	93/80	1	0.013*	74/57	1	0.00005*	19/23	1	0.66
			G/A	40/64	1.86 (1.13–3.05)		17/41	3.13 (1.61–6.07)		23/23	0.83 (0.36–1.91)	
	rs1079727	Codominant	T/T	76/70	1	0.39	48/47	1	0.88	28/23	1	0.24
			C/T	49/53	1.17 (0.71–1.95)		36/37	1.05 (0.57–1.93)		13/16	1.50 (0.60–3.75)	
			C/C	9/15	1.81 (0.74–4.40)		7/9	1.31 (0.45–3.81)		2/6	3.65(0.67–19.85)	
		Dominant	T/T	76/70	1	0.32	48/47	1	0.76	28/23	1	0.18
			C/T-C/C	58/68	1.27 (0.79–2.05)		43/46	1.09 (0.61–1.95)		15/22	1.79 (0.76–4.21)	
		Recessive	T/T-C/T	125/123	1	0.22	84/84	1	0.63	41/39	1	0.15
			C/C	9/15	1.69 (0.71–4.02)		7/9	1.29 (0.46–3.61)		2/6	3.15(0.60–16.58)	
		Overdominat	T/T-C/C	85/85	1	0.75	55/56	1	0.98	30/29	1	0.6
			C/T	49/53	1.08 (0.66–1.77)		36/37	1.01 (0.56–1.82)		13/16	1.27 (0.52–3.11)	
	rs6278	Codominant	C/C	75/75	1	0.83	47/53	1	0.76	28/22	1	0.11
			C/A	53/60	1.13 (0.69–1.85)		40/42	0.93 (0.52–1.67)		13/18	1.76 (0.71–4.36)	
			A/A	8/10	1.25 (0.47–3.34)		7/5	0.63 (0.19–2.13)		1/5	6.36(0.69–58.50)	
		Dominant	C/C	75/75	1	0.57	47/53	1	0.68	28/22	1	0.092
			C/A-A/A	61/70	1.15 (0.72–1.83)		47/47	0.89 (0.50–1.56)		14/23	2.09 (0.88–4.98)	
		Recessive	C/C-C/A	128/135	1	0.73	87/95	1	0.48	41/40	1	0.093
			A/A	8/10	1.19 (0.45–3.10)		7/5	0.65 (0.20–2.14)		1/5	5.12(0.57–45.83)	
		Overdominat	C/C-A/A	83/85	1	0.68	54/58	1	0.94	29/27	1	0.38
			C/A	53/60	1.11 (0.69–1.78)		40/42	0.98 (0.55–1.73)		13/18	1.49 (0.61–3.60)	
	rs6275	Codominant	G/G	45/22	1	0.0013*	30/12	1	0.0036*	15/10	1	0.14
			A/G	72/86	2.44 (1.34–4.44)		52/67	3.22 (1.50–6.90)		20/19	1.43 (0.52–3.94)	
			A/A	20/35	3.58 (1.69–7.57)		13/20	3.85 (1.46–10.12)		7/15	3.21 (0.97–10.69)	
		Dominant	G/G	45/22	1	0.00006*	30/12	1	0.00009*	15/10	1	0.18
			A/G-A/A	92/121	2.69 (1.51–4.79)		65/87	3.35 (1.59–7.03)		27/34	1.89 (0.73–4.87)	
		Recessive	G/G-A/G	117/108	1	0.036*	82/79	1	0.23	35/29	1	0.062
			A/A	20/35	1.90 (1.03–3.48)		13/20	1.60 (0.74–3.43)		7/15	2.59 (0.93-–7.20)	
		Overdominat	G/G-A/A	65/57	1	0.20	43/32	1	0.064	22/25	1	0.68
			A/G	72/86	1.36 (0.85–2.19)		52/67	1.73 (0.97–3.10)		20/19	0.84 (0.36–1.96)	
SLC6A3	rs2652511	Codominant	A/A	70/97	1	0.013*	51/66	1	0.078	19/30	1	0.011*
			A/G	54/39	0.52 (0.31–0.87)		38/24	0.49 (0.26–0.91)		16/15	0.57 (0.23–1.42)	
			G/G	16/9	0.41 (0.17–0.97)		8/8	0.77 (0.27–2.20)		8/1	0.08 (0.01–0.80)	
		Dominant	A/A	70/97	1	0.0037*	51/66	1	0.035	19/31	1	0.037*
			A/G-G/G	70/48	0.49 (0.31–0.80)		46/32	0.54 (0.30–0.96)		24/16	0.41 (0.17–0.96)	
		Recessive	A/A-A/G	124/136	1	0.12	89/90	1	0.98	35/46	1	0.0061*
			G/G	16/9	0.51 (0.22–1.20)		8/8	0.99 (0.36–2.75)		8/1	0.10 (0.01–0.80)	
		Overdominat	A/A-G/G	86/106	1	0.035	59/74	1	0.027	27/32	1	0.6
			A/G	54/39	0.59 (0.36–0.97)		38/24	0.50 (0.27–0.93)		16/15	0.79 (0.33–1.89)	
	rs6347	Codominant	T/T	96/103	1	0.9	63/70	1	0.68	33/33	1	0.45
			T/C	37/35	0.88 (0.51–1.51)		29/25	0.78 (0.41–1.46)		8/10	1.25 (0.44–3.56)	
			C/C	3/3	0.93 (0.18–4.73)		2/3	1.35 (0.22–8.34)		1/0	0.00 (0.00-NA)	
		Dominant	T/T	96/103	1	0.65	63/70	1	0.51	33/33	1	0.84
			T/C-C/C	40/38	0.89 (0.52–1.50)		31/28	0.81 (0.44–1.50)		9/10	1.11 (0.40–3.09)	
		Recessive	T/T-T/C	133/138	1	0.96	92/95	1	0.68	41/43	1	0.23
			C/C	3/3	0.96 (0.19–4.86)		2/3	1.45 (0.24–8.89)		1/0	0.00 (0.00-NA)	
		Overdominat	T/T-C/C	99/106	1	0.65	65/73	1	0.41	34/33	1	0.63
			T/C	37/35	0.88 (0.52–1.51)		29/25	0.77 (0.41–1.44)		8/10	1.29 (0.45–3.66)	
	rs2975226	Codominant	A/A	41/48	1	0.72	29/29	1	0.98	12/19	1	0.33
			A/T	75/71	0.81 (0.48–1.37)		50/49	0.98 (0.51–1.87)		25/22	0.56 (0.22–1.40)	
			T/T	22/21	0.82 (0.39–1.69)		17/18	1.06 (0.46–2.45)		5/3	0.38 (0.08–1.88)	
		Dominant	A/A	41/48	1	0.41	29/29	1	1	12/19	1	0.16
			A/T-T/T	97/92	0.81 (0.49–1.34)		67/67	1.00 (0.54–1.85)		30/25	0.53 (0.21–1.29)	
		Recessive	A/A-A/T	116/119	1	0.83	79/78	1	0.85	37/41	1	0.42
			T/T	22/21	0.93 (0.49–1.78)		17/18	1.07 (0.52–2.23)		5/3	0.54 (0.12–2.42)	
		Overdominat	A/A-T/T	63/69	1	0.54	46/47	1	0.89	17/22	1	0.37
			A/T	75/71	0.86 (0.54–1.38)		50/49	0.96 (0.54–1.69)		25/22	0.68 (0.29–1.60)	
GRIN2B	rs1012586	Codominant	C/C	45/29	1	0.079	28/17	1	0.16	17/12	1	0.44
			G/C	64/79	1.92 (1.08–3.39)		49/59	1.98 (0.97–4.04)		1520	1.89 (0.70–5.12)	
			G/G	25/24	1.49 (0.72–3.09)		16/16	1.65 (0.66–4.13)		9/8	1.26 (0.38–4.20)	
		Dominant	C/C	45/29	1	0.034	28/17	1	0.064	17/12	1	0.28
			G/C-G/G	89/103	1.80 (1.04–3.10)		65/75	1.90 (0.96–3.78)		24/28	1.65 (0.66–4.14)	
		Recessive	C/C-G/C	109/108	1	0.92	77/76	1	0.97	32/32	1	0.83
			G/G	25/24	0.97 (0.52–1.80)		16/16	1.01 (0.47–2.17)		9/8	0.89 (0.30–2.59)	
		Overdominat	C/C-G/G	70/53	1	0.048	44/33	1	0.11	26/20	1	0.09
			G/C	64/79	1.63 (1.00–2.65)		49/59	1.61 (0.89–2.89)		15/20	1.73 (0.71–4.21)	
	rs2268119	Codominant	A/A	46/52	1	0.69	36/37	1	0.68	10/15	1	0.068
			A/T	69/69	0.88 (0.53–1.49)		47/44	0.91 (0.49–1.69)		22/25	0.76 (0.28–2.03)	
			T/T	22/18	0.72 (0.35–1.51)		10/14	1.36 (0.54–3.46)		12/4	0.22 (0.06–0.89)	
		Dominant	A/A	46/52	1	0.51	36/37	1	0.97	10/15	1	0.24
			A/T-T/T	91/87	0.85 (0.52–1.39)		57/58	0.99 (0.55–1.78)		34/29	0.57 (0.22–1.46)	
		Recessive	A/A-A/T	115/121	1	0.46	83/81	1	0.41	32/40	1	0.024
			T/T	22/18	0.78 (0.40–1.52)		10/14	1.43 (0.60–3.41)		12/4	0.27 (0.08–0.91)	
		Overdominat	A/A-T/T	68/70	1	0.9	46/51	1	0.56	22/19	1	0.52
			A/T	69/69	0.97 (0.61–1.56)		47/44	0.84 (0.48–1.50)		22/25	1.32 (0.57–3.05)	
	rs1805502	Codominant	A/A	85/83	1	0.37	59/59	1	0.32	26/24	1	0.72
			G/A	36/49	1.39 (0.82–2.36)		23/32	1.39 (0.73–2.65)		13/17	1.42 (0.57–3.52)	
			G/G	9/7	0.80 (0.28–2.24)		6/3	0.50 (0.12–2.09)		3/4	1.44 (0.29–7.13)	
		Dominant	A/A	85/83	1	0.34	59/59	1	0.55	26/24	1	0.42
			G/A-G/G	45/56	1.27 (0.78–2.09)		29/35	1.21 (0.66–2.22)		16/21	1.42 (0.60–3.34)	
		Recessive	A/A-G/A	121/132	1	0.51	82/91	1	0.26	39/41	1	0.76
			G/G	9/7	0.71 (0.26–1.97)		6/3	0.45 (0.11–1.86)		3/4	1.27 (0.27–6.03)	
		Overdominat	A/A-G/G	94/90	1	0.18	65/62	1	0.24	29/28	1	0.5
			G/A	36/49	1.42 (0.85–2.39)		23/32	1.46 (0.77–2.76)		13/17	1.35 (0.56–3.30)	
	rs7301328	Codominant	G/G	39/35	1	0.86	28/23	1	0.71	11/12	1	0.87
			C/G	72/73	1.13 (0.64–1.98)		48/52	1.32 (0.67–2.60)		24/21	0.80 (0.29–2.19)	
			C/C	27/29	1.20 (0.60–2.40)		18/19	1.29 (0.55–3.00)		9/10	1.02 (0.30–3.44)	
		Dominant	G/G	39/35	1	0.61	28/23	1	0.41	11/12	1	0.76
			C/G-C/C	99/102	1.15 (0.67–1.96)		66/71	1.31 (0.69–2.50)		33/31	0.86 (0.33–2.24)	
		Recessive	G/G-C/G	111/108	1	0.74	76/75	1	0.85	35/33	1	0.75
			C/C	27/29	1.10 (0.61–1.99)		18/19	1.07 (0.52–2.20)		9/10	1.18 (0.43–3.26)	
		Overdominat	G/G-C/C	66/64	1	0.85	46/42	1	0.56	20/22	1	0.59
			C/G	72/73	1.05 (0.65–1.68)		48/52	1.19 (0.67–2.11)		24/21	0.80 (0.34–1.85)	
	rs1806191	Codominant	G/G	98/88	1	0.44	71/62	1	0.32	27/26	1	1
			A/G	37/44	1.32 (0.78–2.24)		22/29	1.51 (0.79–2.89)		15/15	1.04 (0.42–2.54)	
			A/A	3/5	1.86 (0.43–7.99)		2/4	2.29 (0.41–12.94)		1/1	1.04(0.06–17.49)	
		Dominant	G/G	98/88	1	0.23	71/62	1	0.15	27/26	1	0.93
			A/G-A/A	40/49	1.36 (0.82–2.27)		24/33	1.57 (0.84–2.95)		16/16	1.04 (0.43–2.50)	
		Recessive	G/G-A/G	135/132	1	0.46	93/91	1	0.4	42/41	1	0.99
			A/A	3/5	1.70 (0.40–7.27)		2/4	2.04 (0.37–11.44)		1/1	1.02(0.06–16.93)	
		Overdominat	G/G-A/A	101/93	1	0.33	73/66	1	0.25	28/27	1	0.94
			A/G	37/44	1.29 (0.77–2.17)		22/29	1.46 (0.76–2.78)		15/15	1.04 (0.43–2.52)	

^a^* Indicates that *p*-value is statistically significant after FDR correction

**Fig. 2:**
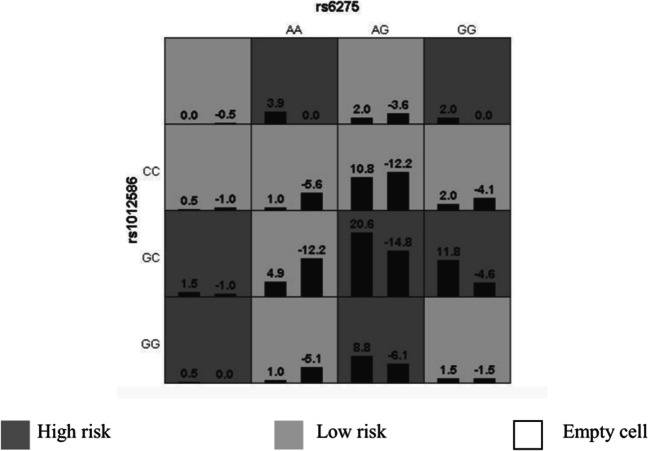
An optimal model exhibits a distinct relationship to ADHD occurrence (*P*<0.001) based on GMDR. The left and right bars in all cells indicate the positive and negative scores, respectively. Dark, light, and no shading indicate high-risk, low-risk, and empty cells, respectively. High- and low-risk cells exhibit different patterns of diverse multilocus dimensions, which indicates epistasis. GMDR, Generalized Multifactor Dimensionality Reduction

For interactions between genes, the optimal interaction model included the two genes present in ADHD. There were different SNPs related to diverse interaction models. We identified an optimal interaction model for the GRIN2B gene rs1012586, and the DRD2 gene rs6275 among the 284 ADHD subjects (Table 3), which had a higher cross-validation agreement (10/10) and validation sample precision (0.6276). These results suggested that GRIN2B genes rs1012586 interacted with the DRD2 gene rs6275 (Fig. 2).

**Table 3:** GMDR models for analyzing multilocus interactions

Model	Training	Testing	*P*	CV
	Bal. Acc.	Bal. Acc.	value	Consistency
1 [rs6275 rs1012586]	0.6556	0.6276	0.011	10/10
2 [rs6275 rs2652511 rs1012586]	0.7060	0.5793	0.010	4/10
3 [rs6275 rs2975226 rs1012586 rs7301328]	0.7906	0.6055	0.011	8/10
4 [rs6277 rs2975226 rs1012586 rs2268119 rs7301328]	0.8614	0.5130	0.172	5/10

## Discussion

This study looked at 13 SNPs in genes involved in the dopaminergic synaptic pathway, as well as interactions between genes linked to ADHD. This research yielded three significant new findings.

In a case-control study, we found a significant association between the DRD2 gene rs6277 and rs6275 and ADHD in Chinese children using codominant, hyper-dominant, and dominant models. Notably, this association was observed only in males. Second, In this case-control study, rs2652511 of the SLC6A3 gene was found to be significantly related to ADHD in Chinese children using the codominant, overdominant, and dominant models. Interestingly, this effect was observed only in females. Third, an exploratory analysis was carried out to assess genetic interactions between the aforementioned genes via GMDR. The GRIN2B gene rs1012586 interacted significantly with the DRD2 gene rs6275.

In a case-control study, we discovered that the DRD2 gene SNPs rs6277 and rs6275 were associated with Chinese children with ADHD. To date, only Finnish [14], East Indian [41], and Chinese Han ancestors [42] have been studied for the association of DRD2 SNPs with ADHD. In a familial study involving 674 children with ADHD, their parents, and siblings from Israel and Europe, an association between the DRD2 gene and ADHD was observed [43]. However, genome-wide association studies conducted on ADHD populations in the USA and Australia did not find any association between the DRD2 gene and ADHD [44]. Given the differences in genetic background among different races, conflicting conclusions have been drawn by various studies. The findings of our study provide support for an association between the DRD2 genes rs6275 and rs6277 with the Chinese population. DRD2 was discovered to be a gender-specific factor in the etiology of ADHD by Nyman et al. [45]. Our study found that the DRD2 gene rs6277 and rs6275 SNPs were associated with ADHD in males only. Previous research has suggested that DRD2 gene expression levels are higher in males compared to females [46], indicating that men may be more responsive to dopamine receptor stimulation and may exhibit higher-risk behaviors. On the other hand, women may require stronger stimuli to produce the same response. These gender differences in DRD2 gene expression levels may contribute to disease risk. It is important to note, however, that these studies are still in the exploratory stage, and further experiments are required to confirm these findings. In particular, research indicates that the rs6277 SNP differs by gender. One study found that in women, the C allele of rs6277 was associated with a higher density of dopamine D2 receptors [47]. However, another study found that men with the T allele (CT or TT) had a higher density of dopamine D2 receptors compared to the CC genotype [48], which differs from females and may be due to differences in gene expression between males and females. In our current study, there was no significant difference in the genotype distribution of the DRD2 gene rs6275 between males and females in children with ADHD. It is important to underscore that the risk associated with ADHD is not solely genetic. Estimates of heritability encompass aspects of gene-environment interactions alongside strictly environmental risks. Research suggests that environmental factors account for approximately 10% to 40% of the variation linked with ADHD [49]. The polymorphism of the DRD2 gene was among the earliest genetic markers discovered to have an association with human behavior, with the A1^+^ genotype correlating with an increased susceptibility to nicotine, alcohol, and illicit substance addiction [50–52]. Several studies have indicated that infants and children exposed to maternal smoking during gestation often exhibit attention difficulties [53] and heightened externalizing behaviors [54]. This serves as a reminder that future research should prioritize investigating the influence of gene-environment interactions on the manifestation of ADHD.

Overall, the DRD2 genes rs6277 and rs6275 may have different effects in different genders, and further research is needed to determine whether there are indeed sex differences. The SNP rs6278 was not linked to ADHD. However, there is no research to back up our findings; Maitra et al. [55] investigated the association of functional DRD2 variants with ADHD in East Indians, and a case-control study revealed that rs6278 was related to ADHD. This study, on the other hand, found no such link in Chinese children with ADHD. This inconsistency may be due to ancestral influences, as genetic patterns differ between ancestral populations [56]. Finally, our findings support the hypothesis that DRD2 is linked to ADHD. More research involving sequencing or SNP technology, as well as longitudinal studies, can aid in understanding and exploring DRD2 in ADHD patients.

SLC6A3 has been extensively suggested to be related to ADHD [24, 57]. The SLC6A3 gene is considered the risk factor for pediatric ADHD [58]. As suggested by Barkley et al., the 9R/10R genotypes were related to ADHD symptoms (impulsivity, externalization, or generalized behavior problems) in children and adolescent populations. However, one latest study queried the relationship between SLC6A3 gene polymorphisms and ADHD and suggested that the SLC6A3 gene in ADHD patients only impacted cognitive flexibility and speed processing. De Azeredo’s study identified that the rs2652511 polymorphism in the SLC6A3 promoter region was the unfavorable factor predicting ADHD occurrence [59]. Our study provides further support for this relationship, demonstrating that it is present only in female students when analyzed for gender dimorphism. However, our study did not show a clear sex difference at the rs2652511 locus. Future studies with larger sample sizes are needed to replicate this finding. In our study, only rs2652511 of the SLC6A3 gene was found to be associated with ADHD, and no association was observed between other loci and ADHD. In contrast, Waldie et al. reported an association between rs6347 and ADHD [18]. However, in our current work, we did not find any suggestive associations. There may be two reasons for this inconsistency. First, this difference may be associated with ancestral impact. Second, it may be associated with age differences in samples, as a recent study revealed changes in the targeting of SLC6A3 genotype regulation with age [28]. This is also an issue to be focused on in further studies.

ADHD is a neurodevelopmental condition that typically commences in childhood and often persists into adulthood. Once perceived as predominantly affecting males, current research corroborates that ADHD is also prevalent among women, albeit sometimes with distinct manifestations [60]. Our study identified sex differences in the expression of several pathogenic loci linked to ADHD, with each locus demonstrating distinct expression variations across sexes. Prior studies have reported higher levels of DRD2 gene expression in males compared to females [46], suggesting a heightened sensitivity to dopamine receptor stimulation in men, which might be linked to riskier behaviors. Additionally, past studies indicate that girls diagnosed with ADHD generally exhibit less hyperactivity compared to boys with ADHD, showing a greater propensity for inattentiveness instead. Montes' research concluded that mood and anxiety disorders tend to affect women more significantly than men, possibly due to women experiencing more mood fluctuations and exhibiting a more emotive disposition compared to men [61]. These findings raise the question of whether inherent physiological and psychological sex differences influence gene expression. This hypothesis necessitates further investigation for validation in future research endeavors.

While these three genes have been independent factors of ADHD pathophysiology in previous studies, this is the first study to report gene-gene interactions of complex genes with ADHD risk and its underlying mechanisms. Complex diseases consist of multiple variants acting together, with smaller effects on individual variants [62]. Multiple genes, as well as SNP, are related to ADHD development. In this work, analysis using the GMDR method suggested that GRIN2B genes rs1012586 were related to the DRD2 gene rs6277. The GMDR interaction is the statistical model. It is hypothesized that biological interactions are less, and variants of the DRD2 gene cause decreased sensitivity and accelerated rate of dopamine uptake and elimination, respectively, which in turn causes a deficit of inter-synaptic neurotransmitters. GRIN2B is a glutamate receptor gene, and glutamate receptor antagonists can interfere with or damage glutamatergic neurons in the prefrontal cortex, which increases inter-synaptic functional protein activity and affects dopamine and norepinephrine reuptake. Although the genotype frequency of the single SNP rs1012586 of the GRIN2B gene was not statistically significant in the ADHD group compared to the controls, a biologically significant interaction of the rs1012586 and rs6277 SNPs cannot be excluded. The gene-gene interactions are ectopic dominant, and the specific biological mechanisms require a functional elucidation of the GRIN2B and DRD2 genes. In our study, several limitations need to be acknowledged. First, we analyzed only 13 SNPs of these three related genes, which provided limited whole-gene coverage. Further exploration by sequencing or genome-wide association studies (GWAS) can help to understand these interactions. Second, after FDR correction, some of the susceptible SNPs of ADHD did not reach significant levels, which may be related to the weak role of the SNP or the sample size, and thus the results of this work must be interpreted cautiously. More studies with large-scale samples are needed. Besides, additional complicated genetic heterogeneity and environmental factors should also be explored.

## Subjects and Methods

### Subjects

Whole-group sampling was conducted to survey all students in grades three to five in seven elementary schools in a region of Xinjiang. A total of 12,800 screening questionnaires were distributed, of which 11,393 were returned. Of these, 528 were excluded because they had more than 50% missing information. Inclusion criteria were as follows: 1) children in grades three to five in elementary school; 2) The Swanson, Nolan, and Pelham, fourth version (SNAP-IV) assessment scale with 6 or more entries, scoring 2 or 3 on a factor as confirmed by a psychiatrist at the attending level or above and meeting the American Psychiatric Association-revised Diagnostic and Statistical Manual of Mental Disorders, fifth edition (DSM-V); 3) The Wechsler Intelligence Scale for Children, fourth edition (WISC-IV) Chinese version, IQ ≥ 85 points; 4) right-handed with normal bare eye vision or corrected vision, no color weakness, voluntary participation, and can adhere to the experiment. Exclusion criteria were as follows: 1) disorders like oppositional defiant, conduct, affective, anxiety, psychotic, and other common mental disorders; 2) a history of traumatic brain injury, neurological diseases, and other serious physical diseases.

The children were further diagnosed by professional physicians based on symptoms, clinical observation, and physical examination. The study finally selected 142 children population experiencing ADHD and 142 normal subjects. The children in both groups had matched ages, grades, and sex. Our study protocols gained approval from the Ethics Committee of the First Affiliated Hospital of Shihezi University, China. Subjects who participated in this work provided written informed consent.

### Assessment

#### Swanson, Nolan, and Pelham Rating Scale-Fourth Version (SNAP-IV)

The SNAP-IV consists of 26 items and is scored on a four-point scale (not at all, just a little, quite a bit, very much). All items fall into one of three subscales: inattention (*n* = 9), oppositional (*n* = 8), and hyperactivity/impulsivity (*n* = 9). Following that, the final results are calculated by averaging the scores from all subscales. Furthermore, items related to hyperactivity/impulsivity and inattention are combined to generate the “pooled ADHD” score [63], with higher scores indicating more severe symptoms. Teachers and parents were invited to complete the SNAP-IV in 15 min on paper or online.

### DNA Extraction, SNP Screening, and Genotyping

To select genes for the dopaminergic synaptic pathway, we first conducted a literature search on ADHD candidate genes in databases, including PubMed, Web of Science, and the China Knowledge Network. We identified candidate genes that were reproducible in different populations, such as DRD2, SLC6A3, COMT, GRIN2B, MECP2, and MAOA. Secondly, we used GeneCards (http://www.genecards.org/) to investigate the relevance of these candidate genes to ADHD, and ranked the top 5 genes based on their relevance scores in these conditions. The top 5 genes were SLC6A3, DRD2, GRIN2B, MAOA, and MECP2. Finally, to enhance the reliability of the study, we used protein interaction networks to identify genes that interact with each other. We used the STRING11.5 analysis tool to analyze the protein interactions of the candidate genes. Based on the evidence for candidate genes and their interactions with other genes, we selected SLC6A3, DRD2, and GRIN2B as the candidate genes for our association study on ADHD.

The steps followed to decipher the 13 candidate SNPs for dopaminergic synaptic pathway genes (DRD2, SLC6A3, GRIN2B) were: Ensembl database (http://asia.Ensembl.org/in-dexHTML/) and NCBI SNP (http://www.ncbi.nlm.nih.gov/snp/) were used to find genes; The functional SNPs were selected from the exon, promoter, 3’UTR and 5’UTR regions of genes; The minimum allele frequencies of these SNPs were evaluated in the 1000 Genomes database (Https://www.ncbi.nlm.nih.gov/variation/tools/1000Genomes/), and the SNPs showing minimum allele frequencies greater than 0.05 were selected. At the same time, Web of Science, PubMed, and Chinese National Knowledge Infrastructure (CNKI) were adopted for searching studies related to candidate gene SNPs, and the SNPs discovered in the study were screened. Finally, 13 SNPs (rs1124491, rs1079727, rs6275, rs6277, rs6278, rs2652511, rs2975226, rs6347, rs1012586, rs1805502, rs1806191, rs2268119, and rs7301328) were selected. These SNPs were analyzed in the SNAP Pairwise LD database for linkage imbalance analysis. SNPs with *R*^2^ > 0.8 in the promoter region were retained, and the pointless SNPs with *R*^2^ > 0.8 in other regions were removed. The genotypic distribution of 13 SNPs was tested by the Hardy-Weinberg equilibrium, which indicated that the samples in this study were representative. Based on the number of detection sites in the final scheme, the primer design was used to determine the information of the final detection sites. The sites to be studied are summarized in Table 4.

**Table 4:** Polymorphisms examined in the present work

Gene	SNP	Location	Allele	MAF (1000Genomes)	*P*
DRD2	rs1124491 rs1079727 rs6275 rs6277 rs6278	Hot SNP Hot SNP Exon Exon 3’UTR	A/G C/T A/G G/A C/A	A = 0.203 C = 0.226 A = 0.473 A = 0.244 A = 0.204	0.657 0.953 0.767 0.940 0.744
SLC6A3	rs2652511 rs2975226 rs6347	Promote Promote Exon	A/G A/T T/C	G = 0.379 T = 0.371 C = 0.298	0.154 0.866 0.623
GRIN2B	rs1012586 rs1805502 rs1806191 rs2268119 rs7301328	Hot SNP 3’UTR Exon Hot SNP Exon	G/C G/A A/G A/T C/G	G = 0.551 G = 0.276 A = 0.247 T = 0.275 C = 0.441	0.143 0.876 0.724 0.809 0.985

This work harvested oral swab specimens from each subject, then the QIAamp DNA Investigation Kit (#DP56504, QIAGEN, Beijing, China) was utilized to extract genomic DNA (gDNA). Using the Sequenom MassARRAY platform at Bio Miao Biological Corporation (Beijing, China), SNP genotyping was performed according to laboratory standard instructions.

### Statistical Analysis

Age factors and intelligence test scores were normally distributed and represented as mean ±standard deviation (SD). Two groups were compared by *t*-test. Sex was a statistical factor, and differences were compared by a Chi-square test. HaploView software was used to analyze whether the genotype frequencies of the target SNPs in the case and control groups met Hardy-Weinberg equilibrium by the Chi-square test. The *P*-value > 0.05 indicated that the Hardy-Weinberg equilibrium was met. SNPStats statistical software was used to analyze genotype frequencies and expressed them as the number of cases. The correlation of variant SNPs with disease risk and the difference in genotype frequencies was analyzed by univariate unconditional logistic regression in different genders. The data were analyzed according to four hypothesis models: dominant, codominant, overdominant, and recessive. All statistics were two-sided tests. After FDR correction, *P*< 0.05 indicated statistical significance. This study utilized GMDR V0.7 software for analyzing interactions between genes.

## Conclusions

This research suggests that dopaminergic synaptic pathway genes (DRD2, SLC6A3) have a significant impact on ADHD susceptibility. Our study suggests that genes involved in the dopaminergic synaptic pathway, such as DRD2 and SLC6A3, play a significant role in ADHD susceptibility. Additionally, the interaction between the GRIN2B and DRD2 genes may also contribute to ADHD susceptibility. Such interactions, however, should be confirmed by other independent or larger samples.
